# Epidemic intelligence needs of stakeholders in the Asia–Pacific region

**DOI:** 10.5365/wpsar.2018.9.2.009

**Published:** 2018-12-18

**Authors:** Aurysia Hii, Abrar Ahmad Chughtai, Tambri Housen, Salanieta Saketa, Mohana Priya Kunasekaran, Feroza Sulaiman, NK Semara Yanti, Chandini Raina MacIntyre

**Affiliations:** a National Centre for Epidemiology & Population Health, Research School of Population Health, Australian National University, Australia.; b School of Public Health and Community Medicine, University of New South Wales, Australia.; c Research, Evidence and Information Programme, Public Health Division, Pacific Community, New Caledonia.; d College of Public Service and Community Solutions, Arizona State University, United States of America.

## Abstract

**Objective:**

To understand the global outbreak surveillance needs of stakeholders involved in epidemic response in selected countries and areas in the Asia–Pacific region to inform development of an epidemic observatory, Epi-watch.

**Methods:**

We designed an online, semi-structured stakeholder questionnaire to collect information on global outbreak surveillance sources and limitations from participants who use epidemic intelligence and outbreak alert services in their work in government and nongovernment organizations in the Asia–Pacific region.

**Results:**

All respondents agreed that it was important to remain up to date with global outbreaks. The main reason cited for following global outbreak news was as an early warning for serious epidemics. Mainstream media and specialist Internet sources such as the World Health Organization (*n* = 54/91; 59%), the Program for Monitoring Emerging Diseases (ProMED)-mail (*n* = 45/91; 49%) and the United States Centers for Disease Control and Prevention (*n* = 31/91; 34%) were the most common sources for global outbreak news; rapid intelligence services such as HealthMap were less common (*n* = 9/91; 10%). Only 51% (*n* = 46/91) of respondents thought that their sources of outbreak news were timely and sufficient for their needs.

**Conclusion:**

For those who work in epidemic response, epidemic intelligence is important and widely used. Stakeholders are less aware of and less frequently use rapid sources such as HealthMap and rely more on validated but less timely traditional sources of disease surveillance. Users identified a need for more timely and reliable epidemic intelligence.

## Introduction

Emerging and re-emerging diseases are significant threats to global health security. The Asia–Pacific region has been the global epicentre for many emerging infectious diseases, including some with pandemic potential. (*1*) The emergence of new diseases such as severe acute respiratory syndrome and avian influenza, the threat of diseases external to the region such as Ebola, and recurring outbreaks of endemic diseases highlight the ongoing threat that infectious diseases pose to national, regional and international health security. (*1*–*4*) The Asia–Pacific region encompasses two World Health Organization (WHO) regions: South-East Asia and the Western Pacific, home to 3.4 billion people, or over 53% of the world’s population. (*5*) The region is one of the most diverse areas in the world in terms of socioeconomic development, geography and geopolitical influence. (*5*) It is also particularly vulnerable to emerging and re-emerging infectious diseases due to several factors including increased population growth and movement, urbanization, globalization, limited access to health care, changes in food trade, land degradation and encroachment on natural habitats and antimicrobial resistance. (*1*, *6*, *7*) This rapidly changing landscape, along with weak health systems, limited health infrastructure, resource constraints (financial, human, technical), geographical isolation and poor population health, challenge countries’ abilities to adequately prevent, detect and respond to public health threats. (*8*–*11*)

The ability to rapidly detect and respond to infectious diseases is critical to global health security. The International Health Regulations, or IHR (2005), provide the legal framework to protect the international community from these threats, requiring Member States to develop core capacities to detect, assess, notify and respond to public health threats and events of national and international concern. (*12*)

IHR (2005) emphasize the importance of incorporating event-based surveillance with traditional systems to detect public health risks. (*12*) Event-based surveillance is “the organized and rapid capture of information about events that are a potential risk to public health.” (*13*) Information can be reported through official or unofficial channels such as media reports, health-care workers and nongovernment organizations. (*14*, *15*) While traditional indicator-based surveillance systems are essential for collecting and analysing information on known diseases, event-based surveillance systems use broad definitions to detect rare or unusual events and are more timely and sensitive. (*13*, *16*, *17*) They are an essential tool for the rapid detection and assessment of events that could pose serious risks to public health.

Increased availability and reliance on the Internet has driven the development and acceptance of event-based Internet surveillance as a key tool and source of epidemic intelligence. (*17*, *18*) This method brings together disparate sources of data from the Internet to provide a comprehensive overview on the current state of global infectious disease events in near real-time for public health action. (*19*) There are three types of event-based Internet surveillance methods for rapid epidemic detection: (1) existing Internet-based surveillance systems and news aggregators that use event-based reporting and syndromic surveillance; (2) search query surveillance using web-based search engines; (3) social media. (*20*)

Understanding countries’ needs to detect and respond to infectious disease risks is relevant to common frameworks such as IHR (2005) and the Asia Pacific Strategy for Emerging Diseases that require cost-effective surveillance tools to coordinate health security activities. There are limited studies on the epidemic intelligence needs of end-users. A review of evaluations of 11 global electronic event-based biosurveillance systems found that evaluations focused on the quantitative analysis of system performance. (*16*) The authors recommended that future evaluations assess the usefulness of systems for public health action for end-users. Stakeholder engagement in all stages of surveillance system development from planning to implementation is important to create a successful and useful system that meets end-users’ needs. (*16*, *21*)

As part of the development of a new epidemic observatory, Epi-watch, we sought to understand the global outbreak surveillance needs of stakeholders involved in epidemic response and surveillance in Australia, Pacific island countries and territories (PICTs), Indonesia and Malaysia. Epi-watch is an epidemic observatory currently in development by Australia’s National Health and Medical Research Council’s (NHMRC) Centre for Research Excellence, Integrated Systems for Epidemic Response (ISER) that monitors and provides critical analysis of global outbreaks and epidemics of public health significance for use by policy-makers, governments and other stakeholders.

The aim of this survey was to understand the global outbreak surveillance needs of stakeholders involved in epidemic response in Australia, PICTs, Indonesia and Malaysia to inform the further development of Epi-watch.

## Methods

A semi-structured stakeholder survey was developed and administered electronically using SurveyMonkey (San Mateo, California, USA) between 27 June 2017 and 9 October. The survey questions pertained to respondents’ employment characteristics (organization location and type, occupation and position level) and global outbreak surveillance sources (automated outbreak alerts, reasons for following outbreak news services, types of sources and services accessed, limitations of outbreak sources, timeliness and adequacy of outbreak news sources, types of journals accessed at least once a month and preferred format to receive information). Responses to questions consisted of pre-defined single and multiple choice options and a free text “other” option.

The survey was piloted in June 2017 on five individuals with infectious disease experience in government and academic institutions in Australia. Minor changes to the survey were made following feedback to improve the consistency and clarity of questions. Pilot participants were not included in the survey sample or results. The final survey was offered in English, French and Bahasa Indonesia. The survey questionnaire was forward-translated into French and Bahasa Indonesia.

We invited participants to complete the survey from the following countries and areas: Australia; PICTs (American Samoa, Cook Islands, Fiji, French Polynesia, Kiribati, Marshall Islands, New Caledonia, Niue, Commonwealth of the Northern Mariana Islands, Samoa, Tokelau, Tonga, Vanuatu); Indonesia; and Malaysia. Our sample was targeted to selected countries so that results would be relevant to inform development of an epidemic intelligence system for use within the region. Malaysia and Indonesia were selected in particular because of ongoing, separate research on epidemic surveillance in the Malay and Indonesian languages.

We used several methods to recruit participants. Eligible participants were those who use epidemic intelligence and outbreak alert services in their work across government and nongovernmental organizations. Purposive and snowball sampling methods were used to select individual participants. Representatives of all PICTs were invited to participate through the Pacific Community (SPC). (*22*) In Australia, participants were identified through the Communicable Diseases Network of Australia, federal and jurisdictional health department web sites, an existing list of public health contacts held by the study team, colleagues and organization web sites. Malaysian and Indonesian participants were identified through ministries of health. Participants were chosen based on their role and field of employment meeting the study inclusion criteria.

The survey was e-mailed to 108 participants from Australia, 13 participants from PICTs, four from Malaysia and three from Indonesia. Participants were asked to forward the survey link to relevant colleagues. Three e-mail reminders to complete the survey were sent to countries with a low response rate to meet our overall target sample size of 88.

In addition to e-mailing eligible participants, a stakeholder workshop was organized by ISER in October 2017 to explore in more depth the outbreak surveillance needs of stakeholders. Workshop attendees were required to complete the survey as a prerequisite for attendance. Eligible attendees at the Communicable Diseases Control Conference in Melbourne, Australia from 27 to 28 June 2017 were also invited to complete the survey.

Responses were downloaded from SurveyMonkey and imported into and analysed using STATA-SE (Version 14.0, StataCorp, College Station, Texas, USA). To calculate proportions, two denominators were used as relevant, total number of responses or respondents. To ensure confidentiality of the respondents and strengthen the analysis, employment characteristic results from PICTs were combined; results from Malaysia and Indonesia (Bahasa Indonesia and Bahasa Malaysia were considered part of a single language group, the Malay language) were also grouped together.

### Ethics

Ethics approvals were obtained from the following committees: University of New South Wales (UNSW) Human Ethics Committee (HC17466), Australian National University Human Research Ethics Committees (2017/517), Malaysia Medical Research and Ethics Committee (NMRR-17–1784–37514), Indonesian Health Research Ethics Committee (LB.02.01/2/KE. 328/2017), Fiji National Health Research Ethics Review Committee (2017.145.MC), Tonga National Health Ethics and Research Committee (310817), and Samoa Health Research Committee (no reference number was allocated). The UNSW ethics approval for conduct of this research was accepted by ministries of health in American Samoa, Cook Islands, French Polynesia, Kiribati, Marshall Islands, New Caledonia, Niue, Commonwealth of the Northern Mariana Islands, Tokelau and Vanuatu.

## Results

There were 96 responses to the survey and a 96% (92/96) completion rate. Of the 128 surveys e-mailed to participants, we received a completed response rate of 72% (92/128). Five responses were excluded because respondents did not meet the study inclusion criteria, completed only the first section of the survey or selected a country from which ethics approval was not obtained, leaving 91 (95%) eligible responses.

### Survey respondent characteristics

Of the 91 respondents, 55% (50/91) worked in organizations based in Australia, 30% (27/91) in organizations in PICTs and 15% (14/91) worked in Malaysia or Indonesia. **Table 1** shows the employment characteristics of survey respondents by region.

**Table 1:** Employment characteristics of survey respondents by country, 2017*

-	Australia		PICTs		Malaysia/ Indonesia	
	*n*	%	*n*	%	*n*	%
Respondents	50	55	27	30	14	15
**Organization type**						
Federal/central government	15	30	13	48	9	64
State/territory government	30	60	4	15	2	14
Local government	3	6	6	22	3	21
International health	0	0	4	15	0	0
Peak body/organization†	2	4	0	0	0	0
**Position level**						
Senior decision-maker‡	17	34	10	37	5	36
Mid-career§	28	56	9	33	5	36
Junior||	3	6	5	19	1	7
Other	2	4	3	11	3	21
Employment type**						
Surveillance, monitoring and control of communicable disease	29	58	22	81	12	86
Planning, prevention and preparedness	17	34	15	56	5	36
General public health	7	14	17	63	6	43
Policy	9	18	8	30	6	43
International emergency response	3	6	12	44	2	14
Domestic emergency response	8	16	2	7	0	0
Acute care	3	6	6	22	1	7
Environmental health	1	2	8	30	0	0
Defence/military	4	8	1	4	0	0
Other	3	6	4	15	1	7

* The total number of respondents by country/region was used as the denominator to calculate percentages separately by country/region.

† Peak body refers to an expert group that provides information, support, advocacy, coordination and strategic guidance to government or nongovernmental organizations.

‡ Senior decision-maker: manages a section/branch/division/head of an organization, has significant and/or final decision-making authority.

§ Mid-career: manages a small team, has some decision-making authority and/or influence.

|| Junior: no management role, has limited authority to make decisions.

** Categories were not mutually exclusive as respondents could select more than one option.

### Importance of global outbreak news

All 91 respondents agreed that it was important to be up to date with global outbreaks. When asked about sources of automated global outbreak alerts (such as Google alerts or Program for Monitoring Emerging Diseases [ProMED]-mail updates), 60% (55/91) reported receiving automated alerts, 18% (16/91) followed outbreak news as required, 15% (14/91) sometimes received automated alerts and 7% (6/91) never got alerts.

The most common reasons for following outbreak news were as an early warning for serious epidemics (91% [83/91]); to inform health system planning, preparedness and response (68% [62/91]); and to inform local surveillance needs (65% [59/91]) (**Table 2**).

**Table 2:** Reasons for following global outbreak news, 2017^*†^

Reasons for following global outbreak news	*n* = 91	%
As an early warning for serious epidemics	83	91
To inform health system planning, preparedness and response	62	68
To inform local surveillance needs	59	65
To inform local clinical and health system needs	40	44
For general interest	35	38
To fulfil IHR (2005) requirements	27	30
For the safety of staff deployed to affected areas	21	23
Outbreak alerts are not relevant for my needs	0	0
Other	4	4

* Categories were not mutually exclusive as respondents could select more than one option.

† Total number of respondents (***n*** = 91) was used as the denominator to calculate percentages to identify the most common reasons for following global outbreak news among all respondents.

### Global outbreak news sources

**Fig. 1:**
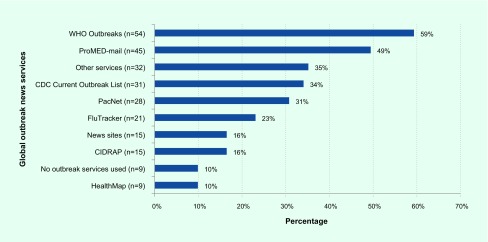
Global outbreak news services used by respondents at least once a month, 2017^*†^

**Fig. 1** shows the proportion of global outbreak information services used by respondents at least once a month. WHO Outbreaks (*23*) was used by 59% (54/91) of respondents and ProMED-mail (*24*) by 49% (45/91).

* Categories were not mutually exclusive as respondents could select more than one option.

† Total number of survey respondents (***n*** = 91) was used as the denominator to calculate percentages to identify the most common service used among all respondents.

CDC = Centers for Disease Control and Prevention; CIDRAP = Centre for Infectious Disease Research and Policy at the University of Michigan, USA; ProMED = Program for Monitoring Emerging Diseases; WHO = World Health Organization.

Other relevant services listed included Outbreak News Today (*25*) (6), Global Public Health Intelligence Network (GPHIN) (*26*) (5), EPICore (*27*) (4), Epi-watch (*28*) (4), Global Incident Map (*29*) (3) and United Nations Dispatch (*30*) (2). In the free text option, the International Biosecurity Intelligence System (25% [2/8]) and the European Centre for Disease Prevention and Control (ECDC) weekly reports and threat assessments (13% [1/8]) were also mentioned.

When asked about other global outbreak news sources, 64% (58/91) of respondents used mainstream media and Internet sources that target health professionals, 49% (45/91) relied on colleagues and 44% (40/91) on health practitioners (**Table 3**). Official sources such as National IHR Focal Points (29% [5/17]), the WHO Event Information Site (24% [4/17]), ECDC (24% [4/17]), the United States Centers for Disease Control and Prevention (USCDC) (18% [3/17]) and networks such as Pacific Public Health Surveillance Network (18% [3/17]) were reported as other sources used by respondents in the free text option.

**Table 3:** Reported timeliness and sufficiency of global outbreak news sources, 2017*

-	Are your sources of global outbreak news timely enough for your needs?						Are your sources of global outbreak news sufficient for your needs?					
Global outbreak news sources^†^	Yes		No		Unsure		Yes		No		Unsure	
	*n*	%	*n*	%	*n*	%	*n*	%	*n*	%	*n*	%
Mainstream media (*n* = 58)	29	50	13	22	16	28	28	48	15	26	15	26
Specialist Internet sources^‡^ (*n* = 58)	36	62	10	17	11	19	32	55	14	24	11	19
Colleagues^‡^ (*n* = 45)	23	51	10	22	11	24	20	44	11	24	13	29
Health practitioners (*n* = 40)	19	48	9	23	12	30	19	48	10	25	11	28
Communicable Diseases Network Australia (CDNA) (*n* = 36)	21	58	4	11	11	31	25	69	6	17	5	14
Social media (*n* = 27)	14	52	9	33	4	15	14	52	9	33	14	15
Other (*n* = 17)	11	65	2	12	4	24	10	59	2	12	5	29

* Total number of responses for each global outbreak news source was used as the denominator to calculate percentages for timeliness and sufficiency for each source separately, as not all respondents used all sources.

† Categories were not mutually exclusive as respondents could select more than one when selecting global outbreak news sources.

‡ One respondent who used these sources reported that timeliness and sufficiency were not relevant to their needs.

Respondents were asked which journals they used at least once a month to access information on global outbreaks and infectious diseases. Multiple responses were allowed. Thirty-seven per cent (34/91) used the USCDC’s Morbidity and Mortality Report, 35% (32/91) used the Bulletin of the World Health Organization, 24% (22/91) used the Western Pacific Surveillance and Response journal, 23% (21/91) used the Australian Department of Health’s Communicable Diseases Intelligence journal and 20% (18/91) used ECDC’s Eurosurveillance journal. Twenty-seven of 91 (30%) respondents did not use any of the journals from the options provided.

### Limitations of global outbreak news

Just over half of respondents, 51% (46/91), thought their usual sources of global outbreak news were timely enough for their needs, 20% (18/91) did not find their sources timely and 29% (26/91) were unsure. Fifty-one per cent (46/91) of respondents thought that their usual sources of global outbreak news were sufficient enough for their needs. Twenty-four per cent (22/91) found their sources were insufficient, and an equal proportion were unsure. One respondent (1/91) reported that timeliness and sufficiency were not personally relevant.

The timeliness and sufficiency of outbreak news sources were cross-tabulated by respondent’s usual sources of global infectious disease outbreak news (**Table 3**). Sixty-two per cent (36/58) of respondents thought that specialist Internet sources such as event-based Internet surveillance systems were timely enough for their needs, and 55% (32/58) found these sources sufficient (**Table 3**).

When asked about the limitations of global outbreak news sources, 42% (38/91) of respondents reported that there was not enough critical appraisal, and 40% (36/91) did not have enough time to read/watch or listen to information. Thirty-two per cent (29/91) of respondents identified that there was not enough information, 30% (27/91) that the sources were not timely enough, and 26% (24/91) that there were too many different sources and did not know which one was best. Twelve per cent (11/91) reported other reasons, such as a delay in or no reporting of events at the country level and lack of local relevance. Nine per cent (8/91) reported no limitations in their sources. Multiple responses were allowed for this question.

### Preferred format to receive global outbreak news

Respondents overwhelmingly preferred e-mail as a mechanism to receive global outbreak news. Eighty-seven per cent (79/91) of respondents selected this option; 7% (6/91) of respondents preferred web sites; 3% (3/91) chose a weekly video presentation; and one each opted for the use of short message service (SMS), social media and other formats. This question did not allow for multiple responses, and feedback from some respondents indicated that they may have had several preferred methods for receiving information, depending on the nature of the outbreak.

A final question asked respondents to provide any other feedback. Answers included needing information for different purposes such as preparation of emergency plans, border health control and advice to traveller consultations; a need to better inform health officials for preparedness, planning and response; and a need for systematized unified surveillance.

## Discussion

Our survey provides insight into the epidemic intelligence needs of a diverse range of stakeholders from across the Asia–Pacific region. There was consensus that timely and easily accessible global outbreak notifications are essential to plan for and respond to public health risks. Respondents’ professional needs are consistent with the key attributes of successful event-based surveillance systems: to be simple, flexible, timely and sensitive. (*15*) With automated alerts being the predominant information-seeking strategy employed by respondents, Internet-based services that provide this function can support the rapid and timely identification of events to limit the spread and severity of disease outbreaks. (*31*)

A limitation of event-based surveillance systems is that new information is not necessarily disseminated efficiently. (*32*) While HealthMap (*33*) is a rapid intelligence source, it was only used by 10% of participants, possibly reflecting low awareness of this resource. Consumers preferred global outbreak alert systems be flexible in the way information is accessed and disseminated. E-mail was identified by respondents as the preferred communication method to receive global outbreak news; however, these needs may change depending on the context of the outbreak and over time (reflecting generational change in the use of communication technology); systems should consider a range of media such as SMS and social media. Communication technologies such as social media can be harnessed for rapid access and dissemination of information to support emergency preparedness and response. (*34*)

The use of mainstream media and specialist Internet sources for global outbreak news is not surprising given the increased accessibility and reliance on the Internet for information and acceptability of event-based Internet surveillance systems. Approximately 65% of initial reports to WHO about infectious disease events come from informal sources such as the Internet. (*35*) A 2017 systematic review of event-based Internet biosurveillance systems identified 50 systems, 37 of which were online and fully functioning at the time. (*36*) Many of these systems use mainstream media as a key source of information. (*17*, *36*) The finding that the same proportion of respondents used both mainstream media and specialist Internet sources for global outbreak news suggests that Internet-based services are not meeting end-users’ needs, and other media sources are required to supplement information leading to duplication of effort.

Timeliness of global outbreak news sources was a limitation identified by 51% of survey respondents. One study explored end-users’ perceptions of the attributes of seven publicly available event-based Internet surveillance systems and found that timeliness scores ranged from 33% to 100%. (*15*) Official sources such as WHO Outbreaks (*23*) and the CDC’s Current Outbreak List (*37*) were more commonly used by respondents over other services such as HealthMap (*33*) but are less timely. Previous studies have documented significant delays in official reporting of outbreaks compared to unofficial reports. (*38*, *39*) Research has identified that the majority of event-based Internet surveillance systems are generated from North America and Europe; few local systems in the Asia–Pacific region and event-based surveillance systems in general are not well understood in developed and developing countries. (*32*, *36*) Increased awareness of the availability and operability of systems providing timely, relevant and reliable information to professionals in the region could address some of these concerns.

Unofficial reports are key sources of information for Internet-based systems, but they can be subject to noise and false alerts, potentially causing unnecessary investigation or alert fatigue among responders. (*18*) Our findings suggest that reliability and accuracy are important considerations in the choice of global outbreak surveillance sources; however, many respondents were unable to identify the best sources to use. WHO Outbreaks (*23*) and ProMED-mail (*24*) were the most commonly accessed sources by many respondents. ProMED-mail is qualitative, but it uses human moderators to review alerts for relevance and accuracy before dissemination, increasing the reliability of reports. (*40*) A service that can provide critical appraisal, including risk assessment within the broader context of the region, could address the need for more reliable information and help facilitate countries’ abilities to assess risks and inform decision-making for the response required.

This study had several limitations. Due to the cross-sectional online survey design, we were unable to monitor trends in responses/behaviour over time, and findings may not be representative because of the snapshot nature of the timing of the survey and possible non-response bias. As we were interested in stakeholder views at a point in time, this design was appropriate. The online nature of the survey meant that questions could not be explored in-depth; however, a free text option was provided for most questions. Limited access to the Internet and computers in remote and resource-constrained areas could have affected the response rate. Compared to positing surveys, this was the most feasible option, and with some of the most remote PICTs participating, we do not believe access was a major barrier. The study employed purposive sampling instead of probability sampling because of the small and highly specialized pool of eligible participants. While this approach ensured participation of professionals from a wide range of backgrounds and levels who use epidemic intelligence, it can create researcher bias because of the judgmental nature of sample selection. Epidemic response is a small and specialized field, so the sample frame from which we could draw was small, making purposive sampling the most appropriate. Limited inclusion of other large Asian countries, differences in participant selection across countries and low numbers of respondents meant that results could not be compared between countries and may not be generalizable to other countries or representative of the whole Asia–Pacific region. Finally, survey versions in languages other than English were not back-translated, which may have affected the quality of these responses. As 11% (*n* = 10) of respondents completed the survey in a language other than English, translation inaccuracies are unlikely to have any impact on the overall validity of the survey. Further research on language-specific needs for epidemic surveillance is warranted.

## Conclusion

For those who work in epidemic response, epidemic intelligence is important and widely used. The choice of sources for global outbreak news varies, and there is less use and awareness of rapid sources such as HealthMap and more reliance on less timely, traditional sources such as WHO and public news media. We identified a need for more timely and reliable epidemic intelligence in the Asia–Pacific region. More effective and efficient sources and methods to deliver user-friendly intelligence to end-users should be explored. There are several global outbreak surveillance systems available; development of a new system should take into consideration how it can integrate into and add value to already established systems within the region.
